# Regioselective *O*-Derivatization of Quercetin via Ester Intermediates. An Improved Synthesis of Rhamnetin and Development of a New Mitochondriotropic Derivative

**DOI:** 10.3390/molecules15074722

**Published:** 2010-07-06

**Authors:** Andrea Mattarei, Lucia Biasutto, Federico Rastrelli, Spiridione Garbisa, Ester Marotta, Mario Zoratti, Cristina Paradisi

**Affiliations:** 1 Department of Chemical Sciences, Università di Padova, via Marzolo 1, 35131 Padova, Italy; 2 Department of Biomedical Sciences, Università di Padova, viale G. Colombo 3, 35121 Padova, Italy; 3 CNR Institute of Neuroscience, viale G. Colombo 3, 35121 Padova, Italy

**Keywords:** regioselective alkylation, mitochondrial targeting, quercetin, rhamnetin, polyphenols

## Abstract

The regioselective synthesis of several quercetin (3,3’,4’,5,7-pentahydroxy flavone) tetraesters bearing a single free OH on 5-C was achieved in good yield by proper choice of reaction conditions using common esterification procedures. Tetracetylated quercetin with the free OH on 7-C was selectively obtained instead via imidazole-promoted deacylation of the corresponding pentaester. Unambiguous structural characterization of the two isomeric tetraacetyl quercetin derivatives was obtained by combined HSQC and HMBC 2D-NMR analysis. These molecules can be used as starting materials for the regioselective synthesis of other derivatives. High yield syntheses of the natural polyphenol rhamnetin (7-*O*-methylquercetin) and of the new mitochondriotropic compound 7-(4-triphenylphosphoniumbutyl) quercetin iodide are reported as examples.

## 1. Introduction

Due to its widespread diffusion in many foodstuffs and to its complex and rich chemistry, quercetin [2-(3,4-dihydroxyphenyl)-3,5,7-trihydroxy-4*H*-chromen-4-one, **1**], is an important and highly representative member of the vast family of natural polyphenols. Such compounds are drawing increasing interest by the scientific community in view of the potentially beneficial effects (anti-inflammatory, anti-ageing, cardioprotective, anticancer, *etc*.) that many of them exhibit *in vitro* (for quercetin see, e.g., [1,2,3,4]). Despite the fact that several natural polyphenols are being exploited as additives in nutritional, cosmetic and over-the counter pharmacological formulations, their activity *in vivo* is difficult to demonstrate due to their low bioavailability [5,6,7]. This in turn is largely a consequence of their high susceptibility to metabolic modifications. Due to the presence of multiple ‒OH’s, polyphenols are ready-made substrates for Phase II conjugative metabolism which rapidly converts them to sulfates, glucuronides and methyl ethers.

One interesting approach to overcome the low bioavailability of polyphenols, so as to test and hopefully exploit their activity *in vivo*, relies on chemical modification of the natural compound aimed at increasing solubility and at slowing down metabolism, while maintaining the capability to regenerate the original molecule. Efforts are obviously concentrating on the protection of the polyphenol hydroxyls [8,9]. Another possibility is the modification of the parent molecule by the introduction of a stable substituent capable of conferring desirable properties. Recently, a charged, membrane-permeant triphenylphosphonium group has been linked to quercetin (at position 3) and resveratrol to produce derivatives targeted to mitochondria, where the polyphenol redox properties either as a pro- or anti-oxidant may be best exploited [10,11,12]. These products join the growing family of mitochondriotropic redox-active compounds [13], the best-known of which may be Mito-Q, a coenzyme Q derivative developed by the pioneering group of Murphy and Smith, currently undergoing clinical trials [14,15], and the plastoquinone-comprising “SkQs” of Skulachev and coworkers [16].

In such a context it is important to be able to modify selectively the various hydroxyls, which are far from equivalent from either the chemical or the biofunctional points of view. Thus, in the case of quercetin, the catechol moiety on the C ring (3’-OH, 4’-OH) is largely responsible for the redox properties of the molecule [17], the 3-OH is a key group for kinase inhibition [18], the 7-OH is chiefly responsible for the weak uncoupling activity [19], and the 5-OH is the least acidic and reactive one, due to intramolecular H-bonding to the carbonyl at 4-C. The literature reports the regioselective acylation of the 3-OH using enzymatic methods [20] or, after protection of the catecholic hydroxyls, chemical reactions [21].

We report here the synthesis and characterization of several ester derivatives of quercetin including pentaesters of a few carboxylic acids with different steric hindrance and selected regioisomers of tetraesters in which the free OH is either on 5-C or 7-C. The regioselective synthesis of tetraesters with a free 5-OH was achieved via esterification of quercetin under controlled conditions. Tetraesters with a free 7-OH can be obtained via selective hydrolysis of pentaester precursors, and we report here the tetraacetylated derivative. These molecules with a single free OH are meant to serve as entry points for the production of other compounds. To illustrate their usefulness we report the synthesis of rhamnetin (7-*O*-methylquercetin), a natural flavonoid and quercetin metabolite which possesses many of the activities of quercetin itself (e.g., it is an inhibitor of mitochondrial NADH oxidase [22]), and of 7-O-(4-triphenylphosphoniumbutyl)quercetin iodide. The latter is a novel mitochondria-targeted compound bearing a free OH at the important 3-position.

## 2. Results and Discussion

### 2.1. Synthesis of 3,3’,4’,5,7-pentaacyl quercetins, of 3,3’,4’,7-tetraacyl quercetins and of 3,3’,4’,5-tetraacetyl quercetin

**Scheme 1:**
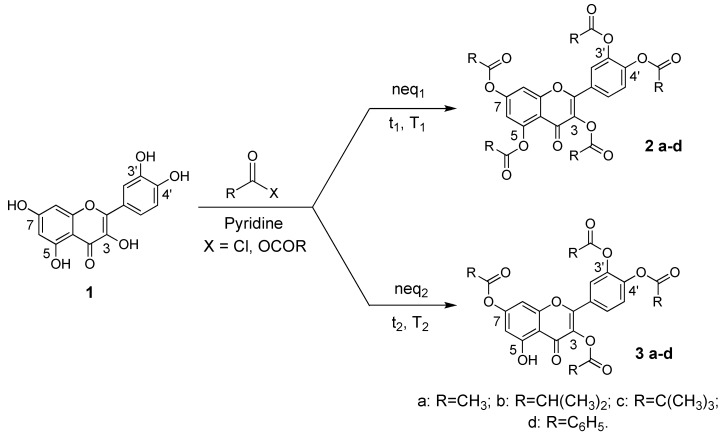
Synthesis of pentaacyl and 3,3’,4’,7-tetraacyl derivatives of quercetin.

Acylation of all five hydroxyl groups of quercetin (**1**) with groups of different steric hindrance was carried out modifying published procedures [8,23] to obtain derivatives **2a–d** in high yield (79–97%) (Scheme 1). By careful control of the reaction conditions, *i.e.* temperature (*T*), type and equivalents (*n_eq_*) of acylating agent and reaction time (*t*), it is possible to stop the acylation at the 3,3’,4’,7-tetraester stage. Reaction yields did not systematically depend on the acyl group. 

Thus, a single isomer of tetraacetyl quercetin was obtained in 75% isolated yield from the reaction with five equiv. of acetic anhydride at room temperature for 3 hours. The product is assigned the structure of 3,3’,4’,7-tetraacetyl quercetin (**3a**) based on ^1^H-NMR analysis. Following literature protocols [24], the assignment of the single free hydroxyl group utilized the narrow peak shape and high chemical shift (12 ppm, 20 mM in CDCl_3_) of the hydroxyl proton [25], and the difference in chemical shifts between the ring protons of the product and the corresponding ones of pentaacetylquercetin (**2a**) (Table 1). The chemical shifts of 2’-H, 5’-H and 6’-H are very similar in **2a** and in **3a**, while those of 6-H and 8-H differ significantly. 

**Table 1:** Chemical shifts (δ) of the aromatic protons of pentaacetyl quercetin (**2a**), 3,3’,4’,7-tetraacetyl quercetin (**3a**) and 3,3’,4’,5-tetraacetyl quercetin (**4**) measured in CDCl_3_. Chemical shift differences (Δδ) relative to **2a** are shown in parentheses.

Compound	δ(H-6)	δ(H-8)	δ(H-5’)	δ(H-6’)	δ(H-2’)
**2a**	6.88	7.33	7.35	7.72	7.69
**3a**	6.60 (-0.28)	6.85 (-0.48)	7.36 (+0.01)	7.75 (+0.03)	7.72 (+0.03)
**4**	6.46 (-0.42)	6.71 (-0.62)	7.24 (-0.11)	7.64 (-0.08)	7.58 (-0.11)

**Scheme 2:**
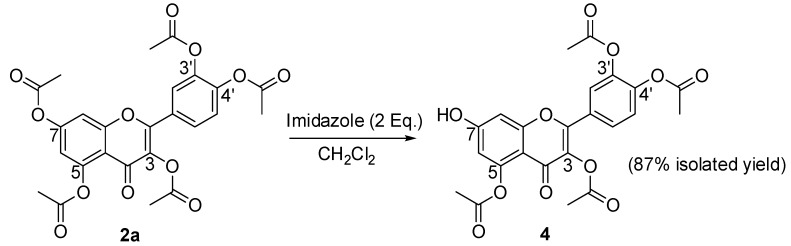
Synthesis of 3,3’,4’,5-tetraacetyl quercetin (**4**).

HSQC (Heteronuclear Multiple-Quantum Correlation) and HMBC (Heteronuclear Multiple-Bond Correlation) 2D NMR analysis was then used to unambiguously establish the structure of **3a** (see Section 2.2). Compounds **3b-d** were synthesized in good yield (70–85%) and characterized by analogous procedures. Details about the specific reaction conditions used are given in the Experimental Section. The good regioselectivity of this reaction can be attributed to the low nucleophilic reactivity of the 5-OH group. The tetraacetyl isomer of quercetin **4** with a free 7-OH was instead prepared with high regioselectivity via imidazole-promoted hydrolysis of pentaester **2a** (Scheme 2). 

Selective deacylation to free the 7-OH group has been reported by Needs and Williamson [26] and Shin *et al.* [27] for daidzen and chrysin, respectively, which have only two hydroxyls. Recently Li *et al*. [28] performed the selective deacylation at 7-OH of pentahexanoyl quercetin using imidazole-catalyzed acyl transfer to an aromatic thiol under basic conditions. In our reaction, without thiols, using 0.2, 1 or 2 equivalents of imidazole produced compound **4** in 14, 44 and 87% isolated yield, respectively. Position 7 is preferentially involved presumably because the 7-OH is the most acidic hydroxyl of quercetin [29]. The assignment of the single free hydroxyl group in **4** is consistent with the observed differences in the chemical shifts of ring protons (Table 1) and was unambiguously established by combined HSQC and HMBC spectra (see Section 2.2). Compound **4** is a useful starting material. Two applications are described below dealing with a new synthesis of rhamnetin (7-*O*-methylquercetin) (**6**) and with the development of a new mitochondria-targeted derivative, 7-*O*-(4-triphenylphosphoniumbutyl) quercetin iodide (**9**).

### 2.2. Spectral assignment of the free hydroxyl position of 3,3’,4’,5-tetraacetylquercetin ***(4)*** and 3,3’,4’,7-tetraacetylquercetin ***(3a)***

**Figure 1:**
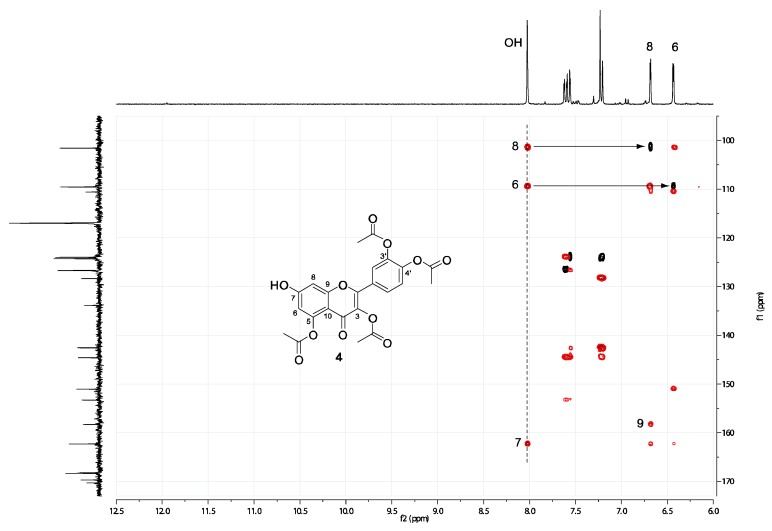
HSQC (black) and HMBC (red) spectra of **4**.

**Figure 2:**
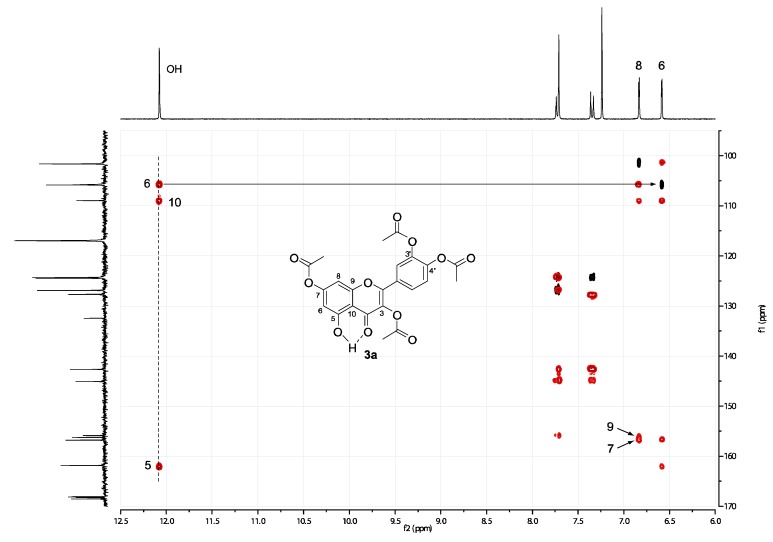
HSQC (black) and HMBC (red) spectra of **3a**.

HSQC and HMBC spectra were obtained in order to confirm the structures of **4** and **3a**. The 2D maps of the relevant spectral regions are shown in Figure 1 and Figure 2 (the ^13^C-NMR spectra displayed as a trace in the indirect dimension were obtained with the UDEFT pulse scheme) [30]. The singlet at 8 ppm in the spectrum of **4** clearly belongs to an exchangeable proton, since it has been found experimentally that trace amounts of water in the solvent mixture change both its lineshape and its position. From the HMBC spectrum (Figure 1, red) it is clear that this proton bears long-range correlations with both 6-C and 8-C (whatever their absolute assignment is) as demonstrated by the superimposed HSQC spectrum (black). This evidence is only compatible with structure **4**.

As a counterproof, in the HMBC spectrum of **3a** (Figure 2), the OH proton appearing at 12.1 ppm bears a long-range correlation with 6-H, thus confirming the proposed structure. In this case the distinction between 6-H and 8-H is unambiguous, since the only proton-bearing carbon seen by the OH in the HMBC is 6-C, whose corresponding 6-H lies at 6.6 ppm as measured from the HSQC. Moreover, the cross peak between 5-C and the signal at 6.6 ppm of the ^1^H spectrum indicates that this resonance indeed belongs to 6-H. Finally, a cross peak between 8-H and 9-C is observed at 156.2 ppm for **3a** and 158.2 ppm for **4**, which further helps to solve the ambiguity in the assignment of 8-H and 6-H. 

### 2.3. Synthesis of rhamnetin (7-O-methylquercetin)

**Scheme 3:**
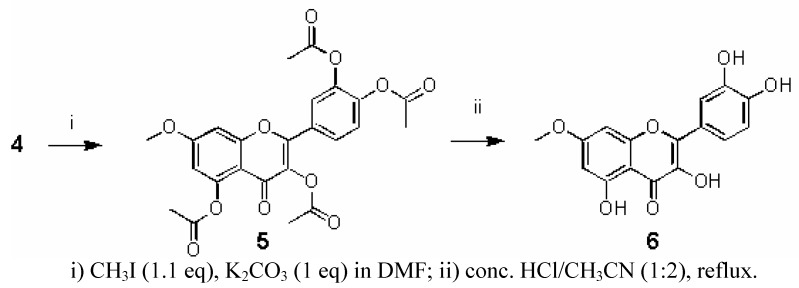
Synthesis of rhamnetin (**6**).

Methylation of **4** followed by hydrolysis under acidic conditions afforded rhamnetin (**6**) in 67% overall yield (Scheme 3). 

The intermediate product **5**, isolated by flash chromatography, contained some *O*-dimethyl derivative(s) (about 20% by NMR analysis). It was used as such, without further purification, in the next step since we found that the final product **6** is easily separated by chromatography from any *O*‑dimethylquercetins. In contrast, it is important to remove any unreacted **4** prior to the hydrolysis step to avoid the formation of quercetin, which is almost impossible to separate from the major product **6** via silica gel flash chromatography. Our procedure, which converts quercetin to rhamnetin in four steps with an overall yield of 46%, is a considerable improvement over a previously reported synthesis also starting from quercetin [21]. The previous procedure requires two consecutive protection steps, the first for the *ortho*-dihydroxyl groups of the B ring, the second for the 3-OH, followed by methylation and two deprotection steps to yield the target compound in 11% overall yield.

### 2.4. Synthesis of 7-O-(4-triphenylphosphoniumbutyl) quercetin iodide ***(9)***

**Scheme 4:**
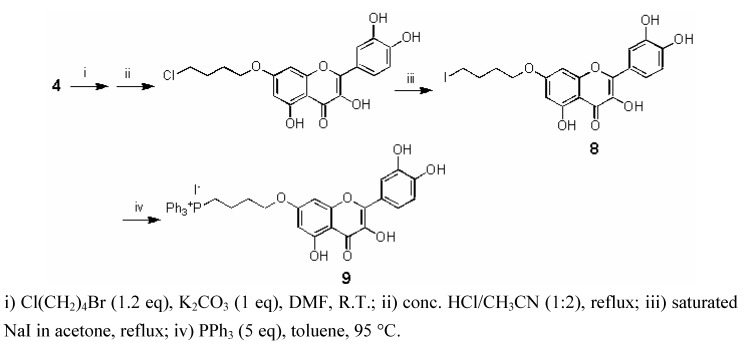
Synthesis of 7-O-(4-triphenylphosphoniumbutyl) quercetin iodide (**9**).

The new mitochondriotropic derivative **9 **was synthesized from **4** in three steps as outlined in Scheme 4.

The synthesis involved one-pot *O*-alkylation of **4** to introduce the chlorobutyl group and complete deacetylation to yield 7-*O*-(4-butylchloro)-quercetin (**7**) in 61% yield. The triphenyl phosphonium (TPP^+^) cation was then introduced via two consecutive nucleophilic substitution reactions first to replace chloride by iodide (giving **8 **in 90% yield) and then to replace iodide by triphenylphosphine (giving **9 **in 73% yield). This two-step procedure allowed us to avoid the high temperatures required by the direct –Cl → –PPh_3_^+^I^−^ conversion, which were found to lead to some product decomposition.

## 3. Experimental

### 3.1. General

Starting materials and reagents were purchased from Aldrich, Fluka, Merck-Novabiochem, Riedel de Haën, J.T. Baker, Cambridge Isotope Laboratories Inc., Acros Organics, Carlo Erba and Prolabo, and were used as received. ^1^H- and ^13^C-NMR spectra were recorded with a Bruker AC 250Fspectrometer or a Bruker Avance DRX 300 spectrometer. Chemical shifts (δ) are given in ppm relative to the residual signal of the solvent (for ^1^H: CDCl_3_: δ = 7.26 ppm, DMSO-d_6_: δ = 2.50 ppm, DMF-d_6_: δ = 8.03 ppm, CD_3_CN: δ = 1.94 ppm; for ^13^C: CDCl_3_: δ = 77.00 ppm, DMSO-d_6_: δ = 39.52 ppm, DMF-d_6_: δ = 29.76 ppm, CD_3_CN: δ = 1.32 ppm). Mass spectra were performed with an Agilent Technologies MSD SL Trap mass spectrometer with ESI source coupled with a 1100 Series HPLC system. TLCs were run on silica gel supported on plastic (Macherey-Nagel Polygram^®^SIL G/UV_254_, silica thickness 0.2 mm), or on silica gel supported on glass (Fluka) (silica thickness 0.25 mm, granulometry 60Å, medium porosity) and visualized by UV detection. Flash chromatography was performed on silica gel [Macherey-Nagel 60, 230-400 mesh granulometry (0.063-0.040 mm)] under air pressure. The solvents were analytical or synthetic grade and were used without further purification. Elemental analyses were performed by the Microanalysis Laboratory of the Dept. of Chemical Sciences of the University of Padova with a Fison EA1108 CHNS Analyzer.

### 3.2. HSQC and HMBC spectra

Spectra were obtained using a Bruker Avance DRX 300 spectrometer equipped with a 5-mm BBO *z*-gradient inverse probe. The experiments were conducted with 20 mM solutions in 1:5 CD_3_CN:CDCl_3_ (chosen to insure solubility during analysis). ^13^C-NMR spectra were obtained with the UDEFT pulse scheme [30]. 

### 3.3. Synthesis of 3,3’,4’,5,7-pentaacylquercetins and 3,3’,4’,7-tetraacylquercetins

*2-(3,4-Diacetoxyphenyl)-4-oxo-4H-chromene-3,5,7-triyl triacetate* (**2a**): Compound **2a** was synthesized from **1** by slightly modifying literature procedures [8,23]. Briefly, quercetin (**1**, 1.00 g, 3.0 mmol, 1 equiv.), acetic anhydride (6.13 g, 60.0 mmol, 20 equiv.) and pyridine (15 mL) were heated to reflux and stirred for 5 h. Ice-water (50 g) was added to the warm mixture. The resulting precipitate was filtered and washed with cold ethyl acetate to afford **2a** as a white solid (1.22 g, 79% yield). ^1^H-NMR (250 MHz, DMSO-d_6_, 25 °C): δ = 2.32 (s, 3H, CH_3_), 2.34 (s, 12H, 4 × CH_3_), 7.16 (d, ^4^*J*_H,H _= 2.2 Hz, 1 H, 6-H), 7.52 (d, ^3^*J*_H,H _= 8.6 Hz, 1 H, 5’-H), 7.64 (d, ^4^*J*_H,H _= 2.2 Hz, 1 H, 8-H), 7.35-7.65 (m, 2H, 2’-H, 6’-H) ppm; ^13^C-NMR (62.9 MHz, CDCl_3_, 25 °C): δ = 170.0, 169.3, 167.9, 167.8, 167.8, 167.8, 156.8, 154.2, 153.7, 150.4, 144.4, 142.2, 134.0, 127.8, 126.4, 123.9, 123.8, 114.7, 113.9, 109.0, 21.2, 21.0, 20.7, 20.5 ppm; MS (ESI-MS): m/z 513, [M+H]^+^; Elemental analysis: calcd. for C_25_H_20_O_12_: C 58.60, H 3.93; found C 58.62, H 3.97.

*4-(3,7-Diacetoxy-5-hydroxy-4-oxo-4H-chromen-2-yl)-1,2-phenylene diacetate* (**3a**): Compound **1** (1.00 g, 3.0 mmol, 1.0 equiv.) was dissolved in CH_2_Cl_2_ (20 mL) and pyridine (5 mL). Acetic anhydride (1.53 g, 15.0 mmol, 5.0 equiv.) was then added dropwise and the mixture was stirred at room temperature for 3 hours. The obtained solution was diluted in CH_2_Cl_2_ (150 mL) and washed with 3 M aq. HCl (3 × 100 mL). The organic layer was then dried over MgSO_4,_ and filtered. The solvent was evaporated under reduced pressure and the residue was purified by silica gel flash chromatography (eluent 9:2:1 CH_2_Cl_2_/hexane/ethyl acetate) to afford **3a** as a yellow solid (1.06 g, 75% yield). ^1^H-NMR (250 MHz, CDCl_3_, 25 °C): δ = 2.34 (m, 9H, 3 × CH_3_), 2.37 (s, 3H, CH_3_), 6.60 (d, ^4^*J*_H,H _= 2.0 Hz, 1 H, 6-H), 6.85 (d, ^4^*J*_H,H _= 2.0 Hz, 1 H, 8-H), 7.36 (d, ^3^*J*_H,H _= 9.0 Hz, 1 H, 5’-H), 7.73-7.77 (m, 2H, 2’-H, 6’-H) ppm; ^13^C-NMR [75.5 MHz, CDCl_3_/CD_3_CN (5:1), 25 °C]: δ = 176.6, 168.5, 168.3, 168.1, 168.0, 161.8, 156.8, 156.3, 155.9, 145.1, 142.7, 132.5, 126.9, 124.5, 124.3, 117.0, 109.0, 105.8, 101.7, 21.3, 20.8, 20.6 ppm; MS (ESI-MS): m/z 471, [M+H]^+^; Elemental analysis: calcd. for C_23_H_18_O_11_: C 58.73, H 3.86; found C 58.65, H 3.85.

*2-(3,4-bis(Isobutyryloxy)phenyl)-4-oxo-4H-chromene-3,5,7-triyl tris(2-methylpropanoate)* (**2b**): Iso-butyric anhydride (4.75 g, 30.0 mmol, 10.0 equiv.) was added dropwise to a solution of **1** (1.00 g, 3.0 mmol, 1.0 equiv.) in pyridine (15 mL). The mixture was heated to reflux and stirred for 2 hours. The obtained solution was diluted in CH_2_Cl_2_ (150 mL) and washed with 3 M aq. HCl (6 × 100 mL). The organic layer was then dried over MgSO_4,_ and filtered. The solvent was evaporated under reduced pressure. The purification of the resulting residue by silica gel flash chromatography (eluent: 5:5:0.25 CH_2_Cl_2_/hexane/ethyl acetate) gave **2b** as a pale yellow solid (1.90 g, 97% yield). ^1^H-NMR (250 MHz, CD_3_CN, 25 °C): δ = 1.23-1.34 (m, 30H, 10 × CH_3_), 2.79-2.94 (m, 5H, 5 × CH), 6.94 (d, ^4^*J*_H,H _= 2.2 Hz, 1 H, 6-H), 7.38-7.41 (m, 2 H, 8-H, 5’-H), 7.73 (d, ^4^*J*_H,H _= 2.0 Hz, 1 H, 2’-H), 7.81 (dd, ^3^*J*_H,H _= 8.8 Hz, ^4^*J*_H,H _= 2.2 Hz, 1 H, 6’-H) ppm; ^13^C-NMR (62.9 MHz, CD_3_CN, 25 °C): δ = 175.9, 175.5, 175.3, 175.1, 174.8, 170.7, 157.9, 155.8, 154.5, 151.4, 145.9, 143.6, 134.8, 128.6, 127.6, 125.2, 124.7, 118.2, 115.5, 110.5, 34.9, 34.8, 34.7, 34.6, 34.5, 19.1, 19.0, 19.0, 18.9, 18.9 ppm; MS (ESI-MS): m/z 653, [M+H]^+^; Elemental analysis: calcd. for C_35_H_40_O_12_: C 64.41, H 6.18; found C 64.45, H 6.22.

*2-(3,4-bis(Isobutyryloxy)phenyl)-5-hydroxy-4-oxo-4H-chromene-3,7-diyl bis(2-methylpropanoate)* (**3b**): Isobutyric anhydride (2.00 g, 12.6 mmol, 4.2 equiv.) was added dropwise to a solution of **1** (1.00 g, 3.0 mmol, 1.0 equiv.) in pyridine (15 mL). The mixture was stirred at 70 °C for 1 hour. The obtained solution was diluted in CH_2_Cl_2_ (150 mL) and washed with 3 M aq. HCl (6 × 100 mL). The organic layer was then dried over MgSO_4,_ and filtered. The solvent was evaporated under reduced pressure and the resulting residue was purified by silica gel flash chromatography (eluent: 5:5:0.25 CH_2_Cl_2_/hexane/ethyl acetate) to afford **3b** as a yellow solid (1.22 g, 70% yield). ^1^H-NMR (250 MHz, CD_3_CN, 25 °C): δ = 1.24-1.30 (m, 24H, 8 × CH_3_), 2.79-2.92 (m, 4H, 4 × CH), 6.61 (d, ^4^*J*_H,H _= 2.0 Hz, 1 H, 6-H), 6.92 (d, ^4^*J*_H,H _= 2.2 Hz, 1 H, 8-H), 7.41 (d, ^3^*J*_H,H _= 8.5 Hz, 1 H, 5’-H), 7.75 (d, ^4^*J*_H,H _= 2.2 Hz, 1 H, 2’-H), 7.82 (dd, ^3^*J*_H,H _= 8.8 Hz, ^4^*J*_H,H _= 2.2 Hz, 1 H, 6’-H) ppm; ^13^C-NMR (62.9 MHz, DMF-d_6_, 25 °C): δ = 176.5, 174.7, 174.3, 174.1, 174.0, 161.4, 157.4, 156.4, 156.0, 145.7, 143.1, 132.3, 127.7, 127.3, 124.9, 108.6, 105.9, 102.3, 34.2, 34.0, 33.9, 18.7, 18.6, 18.5 ppm; MS (ESI-MS): m/z 583, [M+H]^+^; Elemental analysis: calcd. for C_31_H_34_O_11_: C 63.91, H 5.88; found C 63.99, H 5.92.

*2-(3,4-bis(Pivaloyloxy)phenyl)-4-oxo-4H-chromene-3,5,7-triyl tris(2,2-dimethylpropanoate)* (**2c**): Compound **1** (1.00 g, 3.0 mmol, 1.0 equiv.) was dissolved in pyridine (15 mL). Pivaloyl chloride (3.62 g, 30.0 mmol, 10.0 equiv.) was then added dropwise and the mixture was heated to reflux and stirred for 2 hours. The obtained solution was diluted in CH_2_Cl_2_ (150 mL) and washed with 3 M aq. HCl (6 × 100 mL). The organic layer was then dried over MgSO_4,_ and filtered. The solvent was evaporated under reduced pressure. The purification of the resulting residue by silica gel flash chromatography (eluent: 9:1:0.5 CH_2_Cl_2_/hexane/ethyl acetate) gave **2c** as a white solid (1.88 g, 87% yield). ^1^H-NMR (250 MHz, DMF-d_6_, 25 °C): δ = 1.35-1.41 (m, 45H, 15 × CH_3_), 7.27 (d, ^4^*J*_H,H _= 2.2 Hz, 1 H, 6-H), 7.64 (d, ^3^*J*_H,H _= 8.5 Hz, 1 H, 5’-H), 7.69 (d, ^4^*J*_H,H _= 2.2 Hz, 1 H, 8-H), 7.92 (d, ^4^*J*_H,H _= 2.0 Hz, 1 H, 2’-H), 8.01 (dd, ^3^*J*_H,H _= 8.8 Hz, ^4^*J*_H,H _= 2.2 Hz, 1 H, 6’-H) ppm; ^13^C-NMR (62.9 MHz, DMF-d_6_, 25 °C): δ = 176.3, 176.1, 175.7, 175.6, 175.3, 169.8, 157.2, 155.6, 153.8, 151.1, 145.7, 143.2, 128.0, 127.1, 127.1, 124.9, 124.2, 115.0, 114.9, 110.2, 39.3, 39.3, 39.2, 39.1, 39.0, 27.0, 26.9, 26.9, 26.8, 26.7 ppm; MS (ESI-MS): m/z 723, [M+H]^+^; Elemental analysis: calcd. for C_40_H_50_O_12_: C 66.47, H 6.97; found C 66.49, H 7.00.

*2-(3,4-bis(Pivaloyloxy)phenyl)-5-hydroxy-4-oxo-4H-chromene-3,7-diyl bis(2,2-dimethylpropanoate)* (**3c**): Pivalic anhydride (4.47 g, 24.0 mmol, 8.0 equiv.) was added dropwise to a solution of **1** (1.00 g, 3.0 mmol, 1.0 equiv.) in pyridine (15 mL). The mixture was heated to reflux and stirred for 45 minutes. The obtained solution was diluted in CH_2_Cl_2_ (150 mL) and washed with 3 M aq. HCl (6 × 100 mL). The organic layer was then dried over MgSO_4,_ and filtered. The solvent was evaporated under reduced pressure and the resulting residue was purified by silica gel flash chromatography (eluent: 9:1:0.5 hexane/CH_2_Cl_2_/ethyl acetate) to afford **3c** as a pale green solid (1.62 g, 85% yield). ^1^H-NMR (250 MHz, DMF-d_6_, 25 °C): δ = 1.36-1.38 (m, 36H, 12 × CH_3_), 6.79 (d, ^4^*J*_H,H _= 2.0 Hz, 1 H, 6-H), 7.18 (d, ^4^*J*_H,H _= 2.2 Hz, 1 H, 8-H), 7.66 (d, ^3^*J*_H,H _= 8.5 Hz, 1 H, 5’-H), 7.94 (d, ^4^*J*_H,H _= 2.0 Hz, 1 H, 2’-H), 8.0 (dd, ^3^*J*_H,H _= 8.8 Hz, ^4^*J*_H,H _= 2.2 Hz, 1 H, 6’-H) ppm; ^13^C-NMR (62.9 MHz, DMF-d_6_, 25 °C): δ = 176.6, 176.1, 175.7, 175.6, 175.4, 161.5, 157.7, 156.4, 156.0, 146.0, 143.2, 132.5, 127.7, 127.4, 125.0, 124.4, 108.6, 105.9, 102.3, 39.3, 39.3, 39.2, 39.1, 27.0, 26.9, 26.8, 26.7 ppm; MS (ESI-MS): m/z 639, [M+H]^+^; Elemental analysis: calcd. for C_35_H_42_O_11_: C 65.82, H 6.63; found C 65.76, H 6.66.

*2-(3,4-bis(Benzoyloxy)phenyl)-4-oxo-4H-chromene-3,5,7-triyl tribenzoate* (**2d**): Compound **1** (1.00 g, 3.0 mmol, 1.0 equiv.) was dissolved in CH_2_Cl_2_ (20 mL) and pyridine (5 mL). Benzoyl chloride (4.22 g, 30.0 mmol, 10.0 equiv.) was then added dropwise and the mixture was stirred at room temperature for 3 hours. The obtained solution was diluted in CH_2_Cl_2_ (150 mL) and washed with 3 M aq. HCl (3 × 100 mL). The organic layer was then dried over MgSO_4,_ and filtered. The solvent was evaporated under reduced pressure and the resulting residue was purified by silica gel flash chromatography (eluent: 5:5:0.5 CH_2_Cl_2_/hexane/ethyl acetate) to afford **2d** as a white solid (2.4 g, 97% yield). ^1^H-NMR (250 MHz, DMF-d_6_, 25 °C): δ = 7.49-7.83 (m, 15 H, Ar-H), 7.87 (d, ^3^*J*_H,H _= 8.5 Hz, 1 H, 5’-H), 8.01-8.08 (m, 5 H, Ar-H), 8.19-8.29 (m, 8 H, Ar-H), 8.43 (d, ^4^*J*_H,H _= 2.0 Hz, 1 H, 2’-H) ppm; ^13^C-NMR (62.9 MHz, DMF-d_6_, 25 °C): δ = 168.8, 164.9, 164.4, 164.1, 163.9, 163.6, 157.4, 155.5, 154.5, 150.7, 145.6, 143.4, 134.8, 134.7, 134.6, 134.5, 134.2, 133.1, 130.6, 130.5, 130.4, 130.2, 130.2, 129.8, 129.7, 129.4, 129.4, 129.3, 129.1, 128.9, 128.9, 128.6, 128.5, 128.4, 128.4, 127.7, 125.1, 124.6, 115.8, 115.2, 111.0 ppm; MS (ESI-MS): m/z 823, [M+H]^+^; Elemental analysis: calcd. for C_50_H_30_O_12_: C 72.99, H 3.67; found C 72.98, H 3.63.

*4-(3,7-bis(Benzoyloxy)-5-hydroxy-4-oxo-4H-chromen-2-yl)-1,2-phenylene dibenzoate* (**3d**): Compound **1** (1.00 g, 3.0 mmol, 1.0 equiv.) was dissolved in CH_2_Cl_2_ (20 mL) and pyridine (5 mL). Benzoyl chloride (1.77 g, 12.6 mmol, 4.2 equiv.) was then added dropwise and the mixture was stirred at room temperature for 2 hours. The obtained solution was diluted in CH_2_Cl_2_ (150 mL) and washed with 3 M aq. HCl (3 × 100 mL). The organic layer was then dried over MgSO_4,_ and filtered. The solvent was evaporated under reduced pressure. The purification of the resulting residue by silica gel flash chromatography (eluent: 5:5:0.5 CH_2_Cl_2_/hexane/ethyl acetate) gave **3d** as a pale yellow solid (1.55 g, 72% yield). ^1^H-NMR (250 MHz, DMF-d_6_, 25 °C): δ = 7.08 (d, ^4^*J*_H,H _= 2.0 Hz, 1 H, 6-H), 7.49-7.57 (m, 5 H, Ar-H), 7.65-7.75 (m, 6 H, Ar-H), 7.80-7.82 (m, 2 H, Ar-H), 7.88 (d, ^3^*J*_H,H _= 8.5 Hz, 1 H, 5’-H), 8.02-8.06 (m, 4 H, Ar-H), 8.22-8.33 (m, 5 H, Ar-H), 8.44 (d, ^4^*J*_H,H _= 2.0 Hz, 1 H, 2’-H) ppm; ^13^C-NMR (62.9 MHz, DMF-d_6_, 25 °C): δ = 176.6, 164.4, 164.0, 163.9, 163.8, 161.6, 157.5, 156.6, 156.4, 151.1, 145.9, 143.4, 135.0, 134.7, 134.6, 132.7, 130.7, 130.4, 130.2, 130.2, 129.5, 129.4, 129.3, 129.1, 128.6, 128.5, 128.3, 128.2, 127.9, 125.2, 124.8, 108.9, 106.3, 102.8 ppm; MS (ESI-MS): m/z 719, [M+H]^+^; Elemental analysis: calcd. for C_43_H_26_O_11_: C 71.86, H 3.65; found C 71.79, H 3.61.

*Synthesis of 4-(3,5-diacetoxy-7-hydroxy-4-oxo-4H-chromen-2-yl)-1,2-phenylene diacetate* (**4**): A solution of imidazole (0.05 g, 0.78 mmol, 2.00 equiv.) in CH_2_Cl_2_ (5 mL) was added dropwise to a solution of **2a** (0.20 g, 0.39 mmol, 1.00 equiv.) in CH_2_Cl_2_ (10 mL) at –15 °C in an ice/acetone bath. The resulting solution was allowed to warm to room temperature and stirred for 2 hours. The reaction mixture was diluted in CH_2_Cl_2 _(50 mL) and washed with 3 M aq. HCl (3 × 50 mL). The organic layer was then dried over MgSO_4_, and filtered. The solvent was evaporated under reduced pressure. The purification of the resulting residue by silica gel flash chromatography (eluent: CHCl_3_/methanol, 97:3) gave **4** as a white solid (0.16 g, 87% yield). ^1^H-NMR (300 MHz, CDCl_3_/CD_3_CN 5:1, 25 °C): δ = 2.23-2.30 (m, 9H, 3 × CH_3_), 2.34 (s, 3H, CH_3_), 6.48 (d, ^4^*J*_H,H _= 2.2 Hz, 1 H, 6-H), 6.74 (d, ^4^*J*_H,H _= 2.2 Hz, 1 H, 8-H), 7.26 (d, ^3^*J*_H,H _= 9.0 Hz, 1 H, 5’-H), 7.60-7.67 (m, 2H, 2’-H, 6’-H) ppm; ^13^C-NMR [75.5 MHz, CDCl_3_/CD_3_CN (5:1), 25 °C]: δ = 170.31, 169.67, 168.32, 168.2, 162.3, 158.3, 153.3, 151.1, 144.6, 142.6, 133.9, 128.4, 126.7, 124.3, 124.0, 117.0, 110.6, 109.6, 101.6, 21.3, 20.9, 20.7 ppm; MS (ESI-MS): m/z 471, [M+H]^+^; Elemental analysis: calcd. for C_23_H_18_O_11_: C 58.73, H 3.86; found C 58.68, H 3.83.

*Synthesis of 2-(3,4-dihydroxyphenyl)-3,5-dihydroxy-7-methoxy-4H-chromen-4-one* (**6**): A solutionof methyl iodide (0.13 g, 0.95 mmol, 1.1 equiv.) in DMF (10 mL) was added dropwise to a solution of **4** (0.4 g, 0.85 mmol, 1 equiv.) and potassium carbonate (0.12 g, 0.85 mmol, 1.0 equiv.) in DMF (20 mL). The mixture was stirred at –78 °C in a bath of dry ice/acetone for 10 minutes and then allowed to warm and stirred for 12 hours at room temperature. The reaction mixture was diluted in CH_2_Cl_2 _(100 mL) and washed with 3 M aq. HCl (6 × 100 mL). The organic layer was then dried over MgSO_4_, and filtered. The solvent was evaporated under reduced pressure. The purification of the resulting residue by silica gel flash chromatography (eluent: 7:3 CHCl_3_/ethyl acetate) gave a white solid (0.38 g) composed of **5 **and of a dimethylated product in 8:2 ratio, as determined by NMR analysis. ^1^H-NMR (250 MHz, DMSO-d_6_, 25 °C, signals of **5**): δ = 2.27-2.34 (m, CH_3_), 3.93 (s, OCH_3_), 6.87 (d, ^4^J_H,H _= 2.2 Hz, 6-H), 6.30 (d, ^4^*J*_H,H _= 2.5 Hz, 8-H), 7.53 (d, ^3^*J*_H,H _= 9.2 Hz, 5’-H), 7.83-7.90 (m, 2’-H, 6’-H) ppm; MS (ESI-MS): m/z 485, [M+H]^+^, m/z 457, [M’+H]^+^. Without further purification, crude **5** (0.30 g) was added to a solution of acetonitrile (20 mL) and 6 M aq. HCl (10 mL). The resulting solution was stirred and refluxed for 1.5 hours. Then ethyl acetate (100 mL) and water (100 mL) were added. The organic layer was washed with 3 M aq. HCl (3 × 100 mL), dried over MgSO_4,_ and filtered. The solvent was evaporated under reduced pressure and the purification of the resulting residue by silica gel flash chromatography (eluent: 9:1 CHCl_3_/methanol) gave **6** as a bright yellow solid (0.14 g, 67% yield with respect to **4**). ^1^H-NMR (250 MHz, DMSO-d_6_, 25 °C): δ = 3.86 (s, 3H, OCH_3_), 6.35 (d, ^4^*J*_H,H _= 2.0 Hz, 1 H, 6-H), 6.70 (d, ^4^*J*_H,H _= 2.0 Hz, 1 H, 8-H), 6.89 (d, ^3^*J*_H,H _= 8.5 Hz, 1 H, 5’-H), 7.58 (dd, ^3^*J*_H,H _= 8.5 Hz, ^4^*J*_H,H _= 2.0 Hz, 1 H, 6’-H), 7.73 (d, ^4^*J*_H,H _= 2.0 Hz, 1 H, 2’-H) ppm; ^13^C-NMR (62.9 MHz, DMSO-d_6_, 25 °C): δ = 175.9, 164.8, 160.3, 156.0, 147.8, 147.2, 145.0, 136.0, 121.8, 119.9, 115.5, 115.1, 103.9, 97.4, 91.8, 55.9 ppm; MS (ESI-MS): m/z 317, [M+H]^+^; Elemental analysis: calcd. for C_16_H_12_O_7_: C 60.76, H 3.82; found C 60.72, H 3.80.

*Synthesis of 7-(4-chlorobutoxy)-2-(3,4-dihydroxyphenyl)-3,5-dihydroxy-4H-chromen-4-one* (**7**): 1-Bromo-4-chlorobutane (0.62 g, 3.59 mmol, 1.20 equiv) and potassium carbonate (0.413 g, 2.97 mmol, 1.00 equiv.) were added to a solution of **4** (1.39 g, 2.97 mmol, 1.00 equiv.) in DMF (20 mL) under nitrogen and stirred overnight at R.T. The reaction mixture was diluted in CH_2_Cl_2_ (100 mL) and washed with 3 M aq. HCl (3 × 100 mL). The organic layer was then dried over MgSO_4_ and filtered. The solvent was evaporated under reduced pressure. Without further purification, the crude product was added to a mixture of acetonitrile (60 mL) and 3 M aq. HCl (30 mL). The resulting solution was stirred and refluxed for 1 hour, and then ethyl acetate (100 mL) and water (100 mL) were added. The organic layer was washed with 3 M aq. HCl (3 × 100 mL), dried over MgSO_4_, and filtered. The solvent was evaporated under reduced pressure and the purification of the resulting residue by silica gel flash chromatography (eluent: 8:2 toluene/methanol) gave **7** as a bright yellow solid (0.71 g, 61% yield). ^1^H- NMR (250 MHz, DMSO-d_6_, 25 °C): δ = 1.80-2.01 (m, 4H, 2 × CH_2_), 3.66-3.80 (m, 2H, CH_2_), 4.00-4.22 (m, 2H, CH_2_), 6.34 (d, ^4^*J*_H,H _= 2.2 Hz, 1 H, 6-H), 6.70 (d, ^4^*J*_H,H _= 2.0 Hz, 1 H, 8-H), 6.89 (d, ^3^*J*_H,H _= 8.5 Hz, 1 H, 5’-H), 7.57 (dd, ^3^*J*_H,H _= 8.5 Hz, ^4^*J*_H,H _= 2.0 Hz, 1 H, 6’-H), 7.73 (d, ^4^*J*_H,H _= 2.0 Hz, 1 H, 2’-H) ppm; ^13^C-NMR (62.9 MHz, DMSO-d_6_, 25 °C): δ = 175.5, 163.7, 159.9, 155.6, 147.4, 146.8, 144.6, 135.6, 121.5, 119.6, 115.1, 114.8, 103.6, 97.4, 91.9, 67.3, 44.7, 28.3, 25.5 ppm; MS (ESI-MS): m/z 393, [M+H]^+^; Elemental analysis: calcd. for C_19_H_17_ClO_7_: C 58.10, H 4.36; found C 58.18, H 4.41.

*Synthesis of 7-(4-iodobutoxy)-2-(3,4-dihydroxyphenyl)-3,5-dihydroxy-4H-chromen-4-one* (**8**): Compound **7** (0.50 g, 1.27 mmol, 1 equiv.) was added to a saturated solution of NaI in dry acetone (20 mL) and heated at reflux for 20 h. After cooling, the resulting mixture was diluted in EtOAc (100 mL), filtered and washed with water (3 × 30 mL). The organic layer was dried over MgSO_4_ and filtered. The solvent was evaporated under reduced pressure to afford the product in 90% yield after flash chromatography using 7:3 CHCl_3_/acetone. ^1^H-NMR (250 MHz, DMSO-d_6_, 25 °C): δ = 1.71-1.98 (m, 4H, 2 × CH_2_), 3.24-3.43 (m, 2H, CH_2_), 4.06-4.23 (m, 2H, CH_2_), 6.32 (d, ^4^*J*_H,H _= 2.2 Hz, 1 H, 6-H), 6.70 (d, ^4^*J*_H,H _= 2.0 Hz, 1 H, 8-H), 6.89 (d, ^3^*J*_H,H _= 8.5 Hz, 1 H, 5’-H), 7.57 (dd, ^3^*J*_H,H _= 8.5 Hz, ^4^*J*_H,H _= 2.0 Hz, 1 H, 6’-H), 7.73 (d, ^4^*J*_H,H _= 2.0 Hz, 1 H, 2’-H) ppm; ^13^C-NMR (62.9 MHz, DMSO-d_6_, 25 °C): δ = 175.8, 164.0, 160.3, 155.9, 147.7, 147.1, 145.0, 141.5, 135.9, 121.8, 119.9, 115.5, 115.2, 103.9, 92.3, 67.3, 29.5, 29.3, 8.3 ppm; MS (ESI-MS): m/z 485, [M+H]^+^; Elemental analysis: calcd. for C_19_H_17_IO_7_: C 47.13, H 3.54; found C 47.16, H 3.59.

*Synthesis of 7-(4-triphenylphosphoniumbutoxy)-2-(3,4-dihydroxyphenyl)-3,5-dihydroxy-4H-chromen-4-one iodide* (**9**): A mixture of **8** (300 mg, 0.63 mmol, 1 equiv.) and triphenylphosphine (0.825 g, 3.15 mmol, 5 eq.) in toluene (15 mL) was heated at 95°C under argon. After 3 h, the solvent was eliminated at reduced pressure and the resulting yellow solid was dissolved in the minimum volume of dichloromethane (1 mL) and precipitated with diethyl ether (5 × 50 mL). The solvents were decanted after each precipitation. Residual solvent was then removed under reduced pressure to afford compound **6** in 73% yield. ^1^H-NMR (250 MHz, DMSO-d_6_, 25 °C): δ = 1.64-1.80 (m, 2H, CH_2_), 1.88-2.00 (m, 2H, CH_2_), 3.60-3.73 (m, 2H, CH_2_), 4.13-4.20 (m, 2H, CH_2_), 6.28 (d, ^4^*J*_H,H _= 2.0 Hz, 1 H, 6-H), 6.66 (d, ^4^*J*_H,H _= 2.0 Hz, 1 H, 8-H), 6.89 (d, ^3^*J*_H,H _= 8.5 Hz, 1 H, 5’-H), 7.55 (dd, ^3^J_H,H _= 8.5 Hz, ^4^*J*_H,H _= 2.0 Hz, 1 H, 6’-H), 7.72-7.93 (m, 16 H: 15 H = PPh_3_, 1 H = 2’-H) ppm; ^13^C-NMR (62.9 MHz, DMSO-d_6_, 25 °C): δ = 175.7, 163.7, 160.1, 155.8, 147.6, 147.1, 144.8, 135.8, 134.9 [Ph, ^4^*J*(^13^C/^31^P) = 2.8 Hz], 133.4 [Ph, ^3^*J*(^13^C/^31^P) = 10.1 Hz], 130.0 [Ph, ^2^*J*(^13^C/^31^P) = 12.3 Hz], 121.6, 119.7, 118.1 [Ph, ^1^J(^13^C/^31^P) = 86.1 Hz], 115.3, 115.1, 103.8, 97.5, 92.2, 74.1, 28.5, 19.1, 18.2 ppm; MS (ESI-MS): m/z 619, [M]^+^; Elemental analysis: calcd. for C_37_H_32_IO_7_P: C 59.53, H 4.32; found C 59.60, H 4.30.

## 4. Conclusions

Basic procedures and inexpensive and common reagents for esterification and ester hydrolysis were adapted to achieve the regioselective synthesis of quercetin tetraesters bearing the single free OH group either on 5-C or on 7-C. Selective hydrolysis of pentaacetyl quercetin to 3,3’,4’,5-tetraacetyl quercetin (**4**) formed the basis for a new, relatively easy synthesis of rhamnetin (**6**) and for the development of a new quercetin derivative of biomedical interest, the mitochondria-targeted 7-*O*-(4-triphenylphosphoniumbutyl) quercetin iodide (**9**). The cation of **9** is expected to accumulate into cells and mitochondria under the influence of the negative- inside transmembrane potential difference maintained across the cellular and inner mitochondrial membrane. Compound 9 is an isomer of already available 3-*O*-(4-triphenylphosphoniumbutyl) quercetin iodide [10], with which it may be compared in terms of redox reactivity and biological effects. The properties and effects of the two isomers differ indeed significantly (Mattarei, A., *et al*., in preparation), and the comparison will allow an informed choice of the most suitable compound to be tested in experimental models of pathophysiological relevance. The approaches used for the production of these quercetin derivatives may be easily extended to the regioselective modification of other flavonoids.
